# Populations of western North American monkeyflowers accrue niche breadth primarily via genotypic divergence in environmental optima

**DOI:** 10.1002/ece3.9434

**Published:** 2022-10-22

**Authors:** Aeran O. Coughlin, Rachel Wooliver, Seema N. Sheth

**Affiliations:** ^1^ Department of Plant and Microbial Biology North Carolina State University Raleigh North Carolina USA; ^2^ Present address: Department of Biology Duke University Durham North Carolina USA; ^3^ Present address: Department of Biosystems Engineering and Soil Science University of Tennessee Knoxville Tennessee USA

**Keywords:** environmental tolerance, genetic variation, *Mimulus*, niche optimum, specialization, thermal performance curve

## Abstract

Niche breadth, the range of environments that individuals, populations, and species can tolerate, is a fundamental ecological and evolutionary property, yet few studies have examined how niche breadth is partitioned across biological scales. We use a published dataset of thermal performance for a single population from each of 10 closely related species of western North American monkeyflowers (genus *Mimulus*) to investigate whether populations achieve broad thermal niches through general purpose genotypes, specialized genotypes with divergent environmental optima, and/or variation among genotypes in the degree of generalization. We found the strongest relative support for the hypothesis that populations with greater genetic variation for thermal optimum had broader thermal niches, and for every unit increase in among‐family variance in thermal optimum, population‐level thermal breadth increased by 0.508°C. While the niche breadth of a single genotype represented up to 86% of population‐level niche breadth, genotype‐level niche breadth had a weaker positive effect on population‐level breadth, with every 1°C increase in genotypic thermal breadth resulting in a 0.062°C increase in population breadth. Genetic variation for thermal breadth was not predictive of population‐level thermal breadth. These findings suggest that populations of *Mimulus* species have achieved broad thermal niches primarily through genotypes with divergent thermal optima and to a lesser extent via general‐purpose genotypes. Future work examining additional biological hierarchies would provide a more comprehensive understanding of how niche breadth partitioning impacts the vulnerabilities of individuals, populations, and species to environmental change.

## Graphical abstract

Niche breadth, the range of environments that individuals, populations, and species can tolerate, is a fundamental ecological and evolutionary property, yet few studies have examined how niche breadth is partitioned across biological scales. We use a published dataset of thermal performance for a single population from each of 10 closely related species of western North American monkeyflowers (genus *Mimulus*) to investigate whether populations achieve broad thermal niches through general‐purpose genotypes, specialized genotypes with divergent environmental optima, and/or variation among genotypes in the degree of generalization. We found that populations accrue niche breadth primarily via genotypic divergence in environmental optima, and to a lesser extent through general‐purpose genotypes.

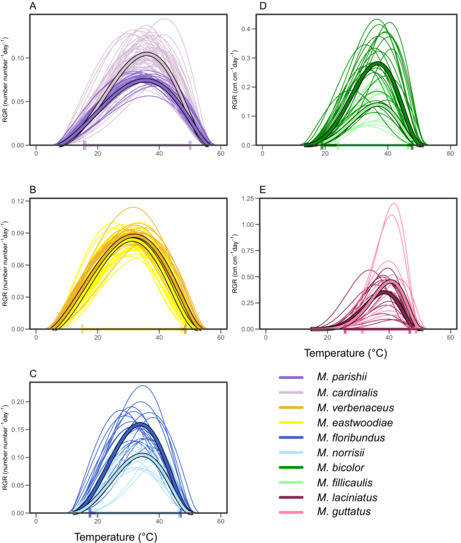

## 1 INTRODUCTION

Niche breadth, the range of environments that individuals, populations, or species can tolerate, is a key ecological and evolutionary property (Carscadden et al., 2020; Futuyma & Moreno, 1988; Hutchinson, 1957; Sexton et al., 2017). Niche breadth is a major axis of rarity (Rabinowitz, 1981), a strong predictor of geographic range size (Sheth et al., 2020; Slatyer et al., 2013), and can influence the vulnerability or resilience of individuals, populations, and species in the face of changing or novel environments (Colles et al., 2009; McKinney, 1997). For instance, species with broad niches may have greater invasion potential (Baker, 1965), and conversely, species with narrow niches may face greater extinction risks under environmental change (Colles et al., 2009; Thuiller et al., 2005). In the strictest sense, niche breadth is the range of conditions across which population growth rates are non‐negative (Hutchinson, 1957), but niche breadth can also be quantified as the range of environments across which individuals, populations, or species maintain high performance (e.g., survival, growth, fecundity, etc.; reviewed in Carscadden et al., 2020; Sexton et al., 2017). Although most studies of niche breadth are at the species level (Carscadden et al., 2020), a species achieves its niche breadth from multiple constituent populations and the individuals within those populations (Bolnick et al., 2003; Carscadden et al., 2020; Sexton et al., 2017). Several recent syntheses have highlighted this hierarchical architecture of niche breadth (Carscadden et al., 2020; Sexton et al., 2017; Sheth et al., 2020; Slatyer et al., 2013), yet few studies have examined how a species' niche breadth is partitioned among populations (Angert et al., 2011; Banta et al., 2012; Hereford, 2017; Kelly et al., 2012; Moritz et al., 2012; Rehfeldt et al., 1999), and in turn, how a population's niche breadth is partitioned among individuals (Araújo et al., 2011; Bolnick et al., 2003; Sultan & Bazzaz, 1993a, 1993b, 1993c).

**FIGURE 1:**
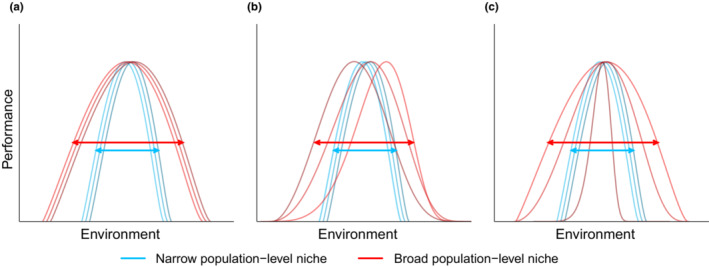
Hypotheses for how genotypes contribute to population‐level niche breadth, where each curve corresponds to a unique genotype and horizontal arrows correspond to the population's niche breadth encompassing the niche breadths of all genotypes combined. (a) General‐purpose genotypes with broad environmental tolerances can lead to a wide population‐level niche; (b) variation in environmental optimum among genotypes can result in a broad population‐level niche; (c) variation in environmental tolerance among genotypes can contribute to population‐level niche breadth.

A population can achieve a broad niche in three main ways. First, a population may consist of general‐purpose genotypes with wide environmental tolerances (Figure 1a; Baker, 1965). For example, studies of two populations of the annual plant *Polygonum persicaria* found that individual genotypes from each population tolerated a broad range of soil moisture and nutrient environments (Sultan & Bazzaz, 1993b, 1993c). Second, specialized individuals with narrow environmental tolerances and divergent environmental optima may contribute to population‐level niche breadth (Figure 1b; Bolnick et al., 2003; Rehfeldt et al., 1999; Roughgarden, 1972). Indeed, there are numerous examples showing that a population's niche breadth is accrued via individual specialization (Araújo et al., 2011; Bolnick et al., 2003). Third, a population may evolve a broad niche via genetic variation for niche breadth among individuals with similar niche optima (Figure 1c; Sexton et al., 2017). Since a broad niche at the individual level is a form of adaptive phenotypic plasticity that stabilizes performance across environments (Pearse et al., 2022; Reusch, 2014; Sexton et al., 2017; Whitlock, 1996), this third mechanism suggests that genetic variation for plasticity can facilitate niche evolution (Colautti et al., 2017). The presence of genetic variation for niche breadth metrics such as climatic tolerance indicates that populations have the potential to evolve broader niches (Hereford et al., 2017; Latimer et al., 2011; Sheth & Angert, 2014a; Vickery, 1972). Though we focus on how niche breadth is partitioned among individuals within populations, these processes also translate to other biological scales such as populations within species and species within clades.

Dissecting the hierarchical nature of niche breadth, and identifying the mechanisms that shape population‐ and species‐level niche breadth are important for predicting vulnerability to climate change (Angert et al., 2011; Carscadden et al., 2020; DeMarche et al., 2018, 2019; Sexton et al., 2017). For instance, a study of the widely distributed copepod *Tigriopus californicus* showed that populations had thermal tolerances that were far narrower than that of the species as a whole (Kelly et al., 2012), suggesting we might underestimate vulnerability to climate change if we assume that population‐level niche breadths encompass the entire species' niche. Nonetheless, numerous experimental studies and meta‐analyses estimate species‐level niche breadth based on a single population (e.g., Deutsch et al., 2008; Pearse et al., 2022), potentially confounding inferences of species vulnerabilities to climate change. Similar issues with underestimating vulnerability could arise if individual‐level niche breadth was only a fraction of a population's niche breadth. Even still, high genetic variation for environmental optimum and breadth within populations could promote adaptation to future climate change (Kellermann et al., 2009).

Here, we reanalyze a published dataset with recently developed hierarchical models to disentangle how niche breadth is partitioned among genotypes of a single population from each of 10 closely related species of western North American monkeyflowers (genus *Mimulus*; Lowry et al., 2019). The dataset is derived from an experiment that Sheth and Angert (2014a) designed to evaluate whether geographically widespread species have broader environmental tolerances than geographically restricted ones (i.e., the niche breadth hypothesis; Brown, 1984; Slatyer et al., 2013). This experiment, which focused on temperature as a key niche dimension that influences photosynthesis, growth, and other physiological processes (Angilletta, 2009), quantified thermal performance curves of five pairs of closely related *Mimulus* species (Table 1) that have contrasting geographic range sizes and climatic niche breadths (Sheth et al., 2014). Previous studies have documented strong effects of temperature on various metrics of performance in *Mimulus* species (Angert, 2006; Paul et al., 2011; Sheth & Angert, 2014a; Vickery, 1974; Wooliver et al., 2020). Sheth and Angert (2014a) found a trend of widespread species having broader thermal performance curves than restricted ones. Since this study only included a single population per species and thus likely underestimated species‐level niche breadth, we focus on examining how the niche breadth of the single population from each of these 10 species is partitioned among genotypes (represented by unique full‐sibling seed families), setting the stage for future work investigating how the niche breadths of multiple populations shape species‐level niche breadth.

**TABLE 1:** Latitude, longitude, elevation, number of families, number of individuals, population‐level thermal breadth (*T*_breadth_), mean family‐level *T*_breadth_, and among‐family variance in thermal optimum (*T*_opt_) and *T*_breadth_ for each focal population of the 10 *Mimulus* species.

Species	Latitude (°N)	Longitude (°W)	Elevation (m)	Number of families	Number of individuals	Population *T*_breadth_ (°C)	Family *T*_breadth_ (°C)	Among‐family variance in *T*_opt_	Among‐family variance in *T*_breadth_	Bayesian *p*‐value
*M. cardinalis* ^a^	33.99329	−116.66267	696	22	691	34.74 (33.61, 35.00)	29.97 (28.48, 31.36)	6.18 (3.37, 9.91)	6.72 (3.12, 11.82)	.67
*M. parishii* ^a^	33.98267	−116.65289	633	50	1327	34.80 (33.78, 35.00)	26.81 (25.61, 28.02)	11.52 (8.18, 15.36)	11.19 (7.68, 15.32)	.41
*M. verbenaceus* ^b^	36.06248	−112.24267	1738	24	762	34.35 (32.78, 35.00)	29.57 (27.96, 30.96)	6.58 (3.72, 10.28)	6.70 (3.52, 10.89)	.44
*M. eastwoodiae* ^b^	36.12917	−109.4545	1743	42	1311	33.63 (32.24, 35.00)	26.89 (25.77, 28.00)	8.12 (5.68, 10.94)	7.95 (5.27, 10.96)	.63
*M. floribundus* ^c^	37.03966	−119.40824	1013	18	242	30.98 (28.57, 33.49)	21.31 (19.91, 22.77)	11.98 (7.01, 18.21)	6.96 (3.32, 11.57)	.54
*M. norrisii* ^c^	36.49858	−118.82067	586	18	268	29.42 (27.14, 31.92)	21.09 (19.70, 22.45)	9.77 (5.6, 14.79)	9.95 (5.58, 15.25)	.71
*M. bicolor* ^d^	38.10399	−120.12173	1549	23	326	30.23 (27.16, 32.96)	18.12 (16.88, 19.37)	12.14 (7.71, 17.22)	5.66 (2.85, 9.36)	.62
*M. filicaulis* ^d^	37.89333	−119.85000	1453	13	179	25.42 (22.16, 28.9)	17.76 (16.19, 19.35)	6.00 (2.22, 11.57)	5.53 (2.10, 10.5)	.52
*M. guttatus* ^e^	37.89333	−119.85000	1453	11	175	20.44 (16.65, 25.59)	13.55 (11.64, 15.43)	3.96 (1.28, 8.75)	3.92 (1.02, 9.69)	.51
*M. laciniatus* ^e^	37.89333	−119.85000	1453	14	170	22.83 (18.9, 27.65)	14.20 (12.84, 15.68)	5.70 (2.37, 11.09)	4.74 (1.87, 8.98)	.43

*Note*: In parentheses below each parameter estimate, 95% credible intervals for population‐ and family‐level *T*_breadth_ and among‐family variance in *T*_opt_ and *T*_breadth_ are shown. Species pairs are grouped by superscript letters (a–e). We used *RGR* in leaf number for species pairs a‐c and *RGR* in stem length for pairs d and e.

We test the relative importance of the following hypotheses and their associated predictions, which are not mutually exclusive. First, if populations achieve broad niches via general‐purpose genotypes (Figure 1a), there should be a positive relationship between family‐level and population‐level niche breadth. Second, if population‐level niche breadth results from specialized genotypes with divergent environmental optima (Figure 1b), there should be a positive relationship between genetic variation in family‐level environmental optimum and population‐level niche breadth. Third, if populations accrue broad niches as a result of variation in niche breadth among genotypes (Figure 1c), there should be a positive relationship between genetic variation in family‐level niche breadth and population‐level niche breadth. Sheth and Angert (2014a) established from their dataset that populations from different monkeyflower species vary widely in thermal performance breadth, with the two populations in each species pair differing by 2.43°C on average. As a result, this dataset allows for a robust test of our hypotheses about how populations accrue niche breadth. Because we control for species phylogenetic relatedness in our analysis, our results are suggestive of how populations (not species) accrue niche breadth.

## 2 MATERIALS AND METHODS

### 2.1 Study system and thermal performance experiment

To address our hypotheses, we used a previously published dataset (Sheth & Angert, 2014a, 2014b) including one population from each of 10 species of *Mimulus* (Phrymaceae), a large genus of wildflowers with approximately 90 species in western North America, and an important model system for research in evolutionary ecology (Beardsley et al., 2004; Grant, 1924; Wu et al., 2008; Yuan, 2019). Due to pronounced effects of temperature on growth and other performance metrics in several *Mimulus* species (Angert, 2006; Vickery, 1974), coupled with notable intra‐ and inter‐specific variation in thermal performance curve parameters (Paul et al., 2011; Sheth & Angert, 2014a; Wooliver et al., 2020), *Mimulus* is an ideal system for studying the partitioning of thermal niche breadth. The study species belong to five pairs in which the two species are closely related (putative sister species or species within the same small subclade; Table 1; Beardsley et al., 2004), yet differ in climatic niche breadth (Sheth et al., 2014) and thermal tolerance (Sheth & Angert, 2014a). Due to a lack of availability of hierarchical models of performance curves, Sheth and Angert (2014a) were unable to fit thermal performance curves to each individual family of each species and were thus limited in their ability to evaluate the hypotheses about niche breadth partitioning that we test in the present study. Sheth and Angert (2014a) attempted to test whether families of the species in each pair with the broader population‐level thermal breadth had wider thermal tolerances than families of the species with narrower population breadth (Figure 1a), and whether species in each pair differed in genetic variation for thermal reaction norms (Figure 1c), but were unable to quantify the degree of divergence in thermal optima among families of each species without family‐level performance curves (Figure 1b).

Briefly, Sheth and Angert (2014a) collected seeds from 20 to 50 individuals of each species either from a single area where both species in each pair co‐occurred or in nearby sites where both species in each pair locally or regionally co‐occurred (Table 1; Figure 2 in Sheth & Angert, 2014a). Seeds resulting from a generation of crosses in the greenhouse (to reduce maternal effects) represented 11–50 unique full‐sibling families per species (Table 1). These seeds were subsequently used in thermal performance experiments that included eight temperature regimes (with daytime temperature ranging from 15 to 50°C) that represent the range of temperatures experienced in the field. To estimate performance, Sheth and Angert (2014a) measured relative growth rate (*RGR*) at early life stages over a 7‐day period in each temperature regime in growth chambers, resulting in a sample size of over 5000 individuals. *RGR* was calculated as the change in leaf number and stem length per initial size per day. While *RGR* is an imperfect proxy for lifetime fitness, it is positively related to flower number in several of the focal species (Weimer A., Sheth S., Unpublished data) and likely influences the probability that pre‐reproductive plants survive to reproduce. For additional details about species selection, field sampling and crosses, and thermal performance experiments, see Sheth and Angert (2014a).

**FIGURE 2:**
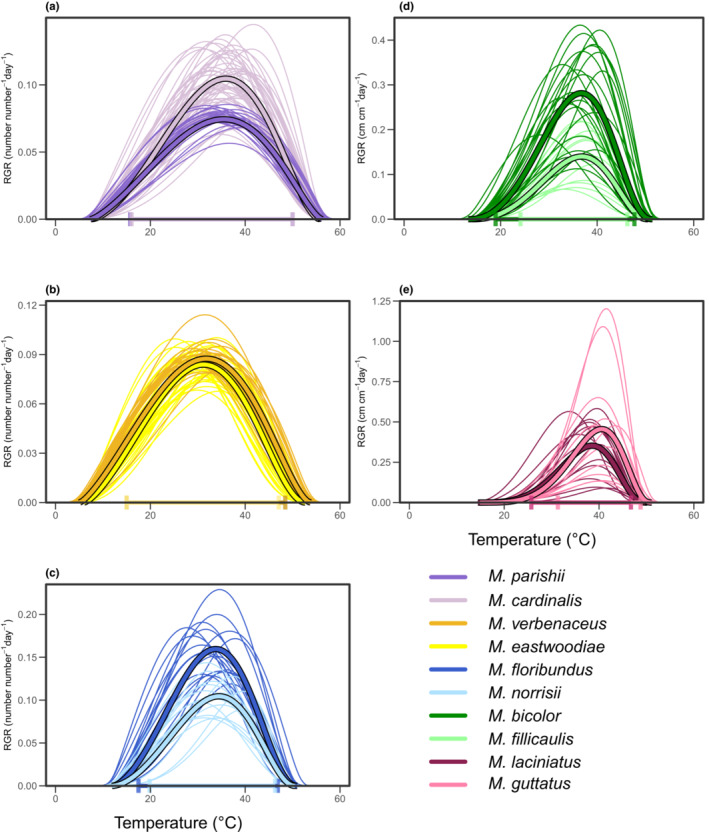
Fitted thermal performance curves for each full‐sibling family (i.e., unique genotype) belonging to five pairs of *Mimulus* species, with darker hues corresponding to the population in each pair with wider thermal breadth (*T*_breadth_) and lighter hues corresponding to the population with narrower *T*_breadth_. The *x*‐axis represents diurnal temperatures from the thermal performance experiment. Bold curves represent the average family‐level curve for each population, while vertical lines on the *x*‐axis correspond to the lower and upper bounds of population‐level thermal breadth (estimated as the difference between the highest upper bound and the smallest lower bound of thermal breadth across all families in the population). In panels (a), (b), and (c), curves were fit to relative growth rate (*RGR*) in leaf number, and in panels (d) and (e), curves were fit to *RGR* in stem length.

Following Sheth and Angert (2014a), we used different *RGR* metrics for different species pairs, depending on which metric captured more variation in growth across temperatures (Table 1). Despite the use of different performance metrics for different species pairs, when fitting thermal performance curve models, we used one model per population of a given species, so *RGR* measured in different units was never included in a single model. Further, the statistical analyses testing our hypotheses, though including all populations in each model, solely used the thermal performance curve parameters *T*_opt_ and *T*_breadth_, both of which are in units of °C, as predictor or response variables, rather than *RGR* itself. This approach does not differ from meta‐analyses of thermal performance that use measures of critical thermal limits derived from different performance metrics across studies (e.g., Bennett et al., 2021; Lancaster & Humphreys, 2020; Sunday et al., 2019).

### 2.2 Thermal performance curve estimation

Thermal performance curves describe the relationship between temperature and a performance metric such as survival, growth, or fecundity (Angilletta, 2009; Huey & Stevenson, 1979). The width of a thermal performance curve provides an estimate of niche breadth based on environmental tolerance (Carscadden et al., 2020; Huey & Stevenson, 1979; Slatyer et al., 2013). We used *RGR* data from Sheth and Angert (2014a, 2014b) to build a thermal performance curve for each family of the focal population of each species (Figures S1–S10). We estimated thermal performance curves with hierarchical Bayesian models that use a derivation of Kumaraswamy's probability density function (R package performr version 0.2; https://github.com/silastittes/performr/tree/zin; Tittes et al., 2019), which accounts for zero‐inflation near the critical thermal limits and has previously been used to estimate thermal performance curves of *Mimulus* species (Querns et al., 2022; Wooliver et al., 2020). We modeled each population separately so that we could simultaneously estimate the thermal performance curves of all families within a given population (i.e., family was included as the grouping factor). One advantage of this approach is that we could use individual *RGR* data rather than averaging *RGR* for each family at each temperature as previous studies (e.g., Angert et al., 2011; Hereford et al., 2017; Sheth & Angert, 2014a) have done to avoid pseudoreplication resulting from the inclusion of multiple, non‐independent *RGR* measurements from replicates of a single family at a single temperature.

Prior to model implementation, *RGR* data were centered around the mean temperature across populations and scaled by population‐specific means to allow the model to more easily explore posteriors. Models of all populations were assigned the same model specifications, including priors for the critical thermal minimum (equivalent to 16°C after back‐transformation) and critical thermal maximum (equivalent to 51°C after back‐transformation), number of iterations (6000), max_tree_depth (12), and adapt_delta (0.95). We standardized these priors across models so that the post hoc analyses of model parameters would not be influenced by the priors. Still, priors were chosen to be weakly informative while maintaining the ability to reject unreasonable parameter values. Adapt_delta controls the step size of the sampler (Stan Development Team, 2019); if adapt_delta is too large, it will constrain the parameter space that the sampler can explore in a given number of steps, while an adapt_delta value that is too small can cause the sampler to explore overly extreme parameter values. Max_treedepth controls the amount of computational effort used for each model iteration (Stan Development Team, 2019). All other model settings were set to the defaults. We assessed model convergence using the potential scale reduction factor ($\hat{R}$, which equaled 1 for all parameters) and reliability of posterior sampling using effective samples (which were at least 700 for family‐level parameters; Gelman et al., 2013). Though this modeling approach is limited to fitting only one type of function to each family instead of allowing for selection among other commonly used thermal performance curve functions (e.g., Gaussian; Angilletta, 2006), the Kumaraswamy function has been shown to fit *Mimulus* thermal performance curves well (Sheth & Angert, 2014a; Wooliver et al., 2020). Further, Bayesian *p*‐values, whose proximity to .5 indicates adequate model fit, were between .41 and .71 for overall models (Table 1).

Our design included multiple full‐sibling families from a single population for each of 10 species, such that for each model iteration we derived thermal breadth (*T*_breadth_) and optimum (*T*_opt_) for each family, along with *T*_breadth_ for each population as a whole. This design, which lacked multiple populations per species, thus did not yield species‐level estimates of *T*_breadth_. Calculating these parameters for each iteration allowed us to obtain their mean and 95% credible interval (i.e., range within which 95% of values fell). Family‐level *T*_breadth_ was estimated as the mean span of temperatures across which the family achieved at least 50% of its performance maximum (Huey & Stevenson, 1979). To prevent extrapolation, if either the lower or upper bound of *T*_breadth_ fell outside of the temperature measurement interval (i.e., below 15°C or above 50°C), it was given the value of the closest measurement temperature (See Table S1 for truncation rates). We estimated population‐level *T*_breadth_ as the difference between the highest upper bound of *T*_breadth_ and the smallest lower bound of *T*_breadth_ across the families of that population (analogous to the “species‐envelope model” in Angert et al., 2011). Then, we estimated *T*_opt_ for each family as the mean temperature at which performance was maximized. In addition, we estimated the mean and 95% credible interval for genetic variation for *T*_opt_ and *T*_breadth_ of each population. Genetic variation for these parameters was calculated as the variance among families of each population for each model iteration. As such, we had one averaged estimate of variance per thermal performance curve parameter per population. We used R version 3.6.1 to fit thermal performance curves (R Core Team, 2019).

### 2.3 Hypothesis testing

Similar to the paired *t*‐test approach in Sheth and Angert (2014a) to compare *T*_breadth_ of widespread and restricted species, we implemented linear mixed effects models with species pair included as a random effect to account for shared evolutionary history between populations in each pair. Because Sheth and Angert (2014a) chose species pairs that were either sister species or species within the same small subclade, including species pair as a random effect in each model was sufficient to account for phylogenetic non‐independence of populations within each pair. Specifically, we allowed each species pair to have a unique intercept, with slopes remaining constant for all pairs. Slope values would thus indicate the associations shared between each predictor variable and population‐level *T*_breadth_. First, we examined the effect of family‐level *T*_breadth_ on population‐level *T*_breadth_, with a positive effect supporting the hypothesis that populations with greater niche breadth are composed of generalist genotypes with broad environmental tolerance (Figure 1a). Second, we modeled population‐level *T*_breadth_ as a function of genetic variation for *T*_opt_, with a positive effect supporting the hypothesis that populations achieve broad environmental tolerance through greater genetic variation in *T*_opt_, indicative of specialized genotypes with divergent optima. Third, we examined the effect of genetic variation in family‐level *T*_breadth_ on population‐level *T*_breadth_, with a positive effect supporting the hypothesis that broad population‐level niches are achieved through greater genetic variation for environmental tolerance. For each model, we evaluated whether the slope describing the relationship between the predictor variable and population *T*_breadth_ was significantly >0 at *α* = .1. We used the MuMIn package v. 1.43.17 (Bartoń, 2020) to calculate marginal *R* ^2^ (i.e., the variance explained by fixed effects) and conditional *R* ^2^ (i.e., the variance explained by both fixed and random effects; Nakagawa et al., 2017). To determine which of the three predictors of population‐level *T*_breadth_ was strongest, we re‐implemented linear mixed effects models with predictor and response variables centered and scaled (as described in Ware et al., 2019). Slope coefficients from these standardized models are comparable and would indicate which predictors were weakly related to population‐level *T*_breadth_ (slope coefficient closer to 0) or strongly related to population‐level *T*_breadth_ (slope coefficient closer to −1 or +1). All analyses associated with hypothesis testing were conducted with the lme4 package (Bates et al., 2015) in R version 4.1.0 (R Core Team, 2021), and the data and code associated with these analyses are available at https://github.com/emcoughlin/mimulus‐breadth‐partitioning.

## 3 RESULTS

**FIGURE 3:**
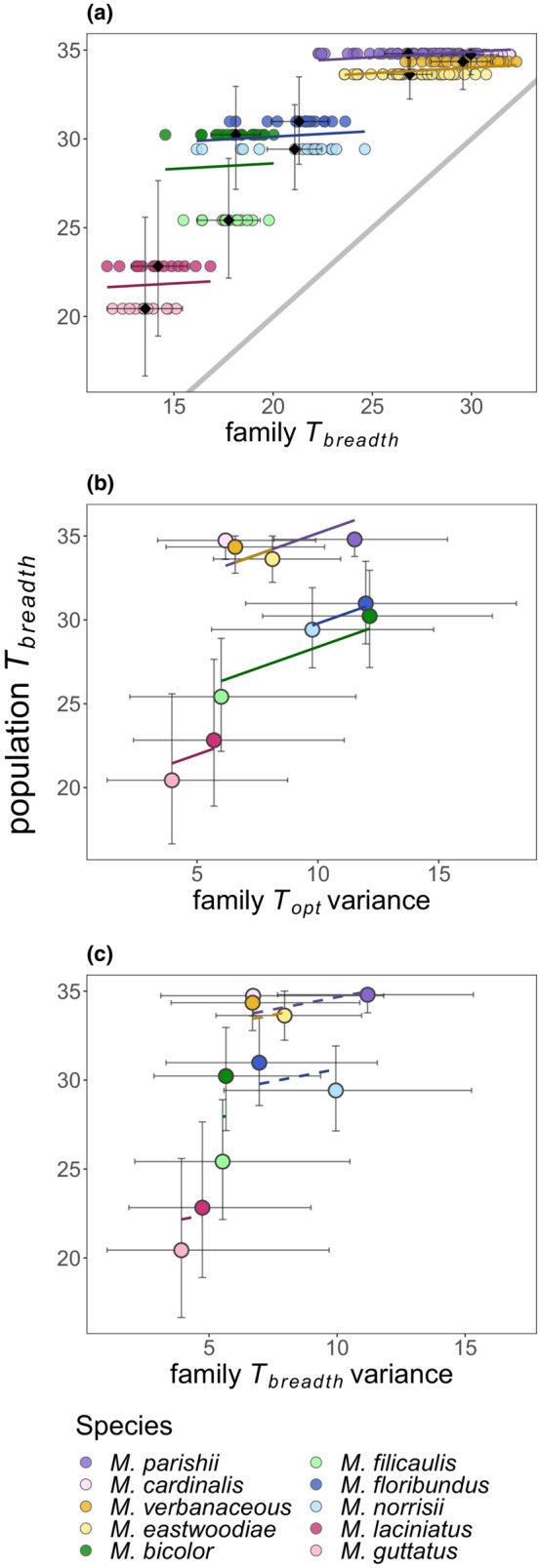
Relationships between population‐level thermal breadth (*T*_breadth_) and (a) family‐level *T*_breadth_, (b) among‐family variance in thermal optimum (*T*_opt_), and (c) among‐family variance in *T*_breadth_ across species pairs. Black diamonds in panel (a) indicate mean family‐level *T*_breadth_, and filled circles represent *T*_breadth_ values for each family. In all panels, species pairs are grouped by color. The species with wider population‐level *T*_breadth_ within each pair is indicated by a darker hue, while the species with narrower population‐level *T*_breadth_ is indicated by a lighter hue. Mean values with 95% credible intervals are shown in all panels. The gray line in panel (a) shows a 1:1 relationship between family‐ and population‐level *T*_breadth_. Solid‐colored lines show fitted regression slopes that are >0 (*p* < .1), and dashed‐colored lines show slopes that are not >0 (*p* > .1). Because species pair was modeled as a random effect with variable intercepts to account for phylogenetic non‐independence, separate regression lines with unique intercepts are shown for each pair.

In support of the hypothesis that population‐level niche breadth is achieved through general‐purpose genotypes, we found that family‐level *T*_breadth_ is positively related to population‐level *T*_breadth_ (Figure 3a). On average, family‐level *T*_breadth_ represented ~60%–86% of the population‐level *T*_breadth_ of each species (Table 1), such that every 1°C increase in family‐level *T*_breadth_ resulted in a 0.062°C (±0.034 SE) increase in population‐level *T*_breadth_ (*p* = .066; Figure 3a). However, family‐level *T*_breadth_ explained <1% of the variation in population‐level *T*_breadth_ (marginal *R* ^2^ = .005). Instead, species pair explained the majority of variation in population‐level *T*_breadth_ (conditional *R* ^2^ = .955), suggesting that phylogenetic relatedness is more powerful than family‐level *T*_breadth_ in predicting population‐level *T*_breadth_.

There was also support for the hypothesis that divergence in *T*_opt_ contributes to population‐level niche breadth (Figure 3b). For every unit increase in among‐family variance in *T*_opt_, there was a 0.508°C (±0.213 SE) increase in population‐level *T*_breadth_ (*p* = .070; Figure 3b). The standard deviation in *T*_opt_ (i.e., the square root of variance in *T*_opt_ from Table 1) ranged from 1.99 to 3.48°C across populations. Similar to the first model, there was a high conditional *R* ^2^ relative to marginal *R* ^2^ (marginal *R* ^2^ = .087, conditional *R* ^2^ = .931), indicating that population‐level *T*_breadth_ is driven by variation among species pairs.

Inconsistent with the hypothesis that population‐level niche breadth is accrued through genetic variation in breadth, there was no relationship (slope = 0.275 ± 0.485 SE, *p* = .597) between among‐family variation in *T*_breadth_ and population‐level *T*_breadth_ (Figure 3c). As in the previous two models, species pair also explained the majority of variance in population‐level *T*_breadth_ (marginal *R* ^2^ = .014, conditional *R* ^2^ = .841).

Standardized models indicated that relative to family‐level *T*_breadth_ and among‐family variation in *T*_breadth_ (standardized slopes = 0.083 ± 0.045 SE and 0.120 ± 0.211 SE, respectively), genetic variation for family‐level *T*_opt_ had the greatest effect on population‐level *T*_breadth_ (standardized slope = 0.291 ± 0.122 SE).

## 4 DISCUSSION

In this study, we re‐analyzed previously published thermal performance data for populations of 10 western North American monkeyflower species (Sheth & Angert, 2014a) to understand whether populations achieve broad niches through general‐purpose genotypes (Baker, 1965), specialized genotypes with divergent environmental optima (Bolnick et al., 2003), and/or variation among genotypes in the degree of generalization (Sexton et al., 2017). Consistent with the hypothesis that populations accrue niche breadth via general‐purpose genotypes, family‐level thermal breadth had a positive effect on population‐level breadth across the 10 *Mimulus* species. However, we found stronger support for the hypothesis that divergent environmental optima contribute to population‐level breadth, where populations with greater genetic variation for thermal optimum had greater thermal breadth. Since genetic variation for family‐level thermal breadth was unrelated to population‐level thermal breadth, our results did not support the hypothesis that genetic variation for niche breadth contributes to population‐level breadth. Together, these findings suggest that populations of the 10 focal *Mimulus* species achieve thermal breadth primarily through specialization in thermal optimum and to a lesser extent general‐purpose genotypes. This study sheds light on how population‐level niche breadth is partitioned among individuals and reveals that individual niche breadth represents a substantial portion of population niche breadth in our study populations (Hereford et al., 2017; Sultan & Bazzaz, 1993b). Below, we interpret these findings in light of the focal hypotheses, compare our approach and results with those from Sheth and Angert (2014a), and describe some caveats that might limit the inferences in our study. Ultimately, our findings shed light on one level of the hierarchical partitioning of niche breadth, a first step for future studies of niche breadth partitioning among populations and species.

Our findings suggest that genotypes with both broad environmental tolerance and divergent environmental optima can together lead to a wide population‐level niche. This contrasts from previous work documenting the prevalence of either general purpose genotypes or individual specialization in shaping population‐level niche breadth. For example, a series of experiments with the annual plant *Polygonum persicaria* along multiple niche axes showed that individual genotypes can tolerate a broad range of environments (Sultan & Bazzaz, 1993a, 1993b, 1993c). Similarly, a study based on one population from each of 11 species of jewelflower (genus *Streptanthus*) showed that genotypes of species with broader hydrological niches maintained more stable fitness across soil moisture treatments relative to those from species with narrower hydrological niches (Pearse et al., 2022). In contrast, most empirical work highlighting the role of individual specialization in shaping population‐level niche breadth focuses on resource use metrics such as dietary niche breadth (Araújo et al., 2011; Bolnick et al., 2007). Though we speculate that the relative contributions of genotype‐level thermal tolerance and divergence in thermal optima among genotypes could vary by species pair, this was not in the scope of our study and merits future work. Though there is a growing number of studies of how climatic tolerances are partitioned among populations within single species (e.g., Angert et al., 2011; Kelly et al., 2012; Rehfeldt et al., 1999; Sasaki & Dam, 2019), fewer studies have examined how population‐level niche breadth quantified as environmental tolerance is partitioned among individuals.

Our results based on thermal performance curves fit to each family provide unique insights into niche breadth partitioning relative to the original study by Sheth and Angert (2014a). First, while Sheth and Angert's (2014a) calculation of population‐level breadth represented an average across families, ours encompassed the maximum achievable breadth across families, as conceptualized in Figure 1. As a result, the relative ranking of population niche breadth for *M. guttatus* and *M. laciniatus* was flipped. These inconsistent results across different methodologies suggest that further work is needed to develop performance curve models that better represent the hierarchical nature of niche breadth. Second, perhaps due to the aforementioned difference in the calculation of population breadth, Sheth and Angert (2014a) found that *M. verbenaceus*, *M. floribundus*, and *M. guttatus*, the species in each pair with the broader population‐level thermal performance curve, had greater family‐level thermal breadth than their counterparts (*M. eastwoodiae*, *M. norrisii*, and *M. laciniatus*, respectively), but the species in the remaining two pairs did not differ in mean family‐level breadth. Their findings provide partial support for the hypothesis that general‐purpose genotypes contribute to broad population‐level tolerance. In contrast, we found a positive relationship between family‐ and population‐level thermal breadth across all populations while taking into account the random effect of pair (Figure 3a). Third, Sheth and Angert (2014a) quantified genetic variation for thermal performance breadth as the among‐family variance in the slopes of *RGR* between both 15 and 20°C and 45 and 50°C, and showed that in all species pairs, the species with broader population‐level tolerance had greater genetic variation for thermal reaction norms at both temperature extremes relative to the species with narrower population‐level tolerance. In contrast, we quantified genetic variation for thermal breadth as the among‐family variance in the width of family‐level thermal performance curves and found no relationship between genetic variation for thermal breadth and population‐level thermal breadth. Jointly, the results from the current study and Sheth and Angert (2014a) reveal that genetic variation for thermal reaction norms at temperature extremes, rather than overall breadth of the performance curve, may facilitate the evolution of broad population‐level thermal tolerance.

This study has a few important caveats that could impact our inferences of niche breadth partitioning among genotypes within a single population of each focal *Mimulus* species. First, because the dataset only included a single population per species, we were unable to partition niche breadth among populations of each species. Future work that considers the role of local adaptation in restricting the niche breadths of populations across the geographic range of each species is needed, since populations could occupy niches that are far narrower than the species‐level niche (Kelly et al., 2012). The inclusion of only one population per species also prevents us from clearly distinguishing effects that arise from species‐level differences from those that stem from population‐level differences. Yet, in many meta‐analyses and comparative studies of thermal or hydrological performance (e.g., Bennett et al., 2021; Lancaster & Humphreys, 2020; Pearse et al., 2022; Sunday et al., 2019), data are generally collected at the population level and given the label of species level without us ever truly knowing if each data point is representative of each respective species. We have no a priori reason to expect that a single population represents the niche breadth of the entire species, nor do we have reason to believe that these populations are outliers given that none of them were collected at the geographic or climatic margins of the species range or niche. Despite the potential for unmeasured species‐level traits that contribute to the observed interspecific variation in thermal breadth, family‐level breadth, and among‐family variance in thermal optimum were nonetheless positively related to the thermal breadth of the focal population of each species. Second, with a sample size of only 10 populations, our power to detect support for our hypotheses was likely limited. Future studies involving organisms that are amenable to larger sample sizes would shed further light into the relative roles of general‐purpose genotypes, specialized genotypes with divergent environmental optima, and variation among genotypes in the degree of generalization in shaping niche breadth at various biological scales. Third, we used *RGR* over the short time frame of 7 days as a measure of performance, yet *RGR* over a 1‐week period may not accurately reflect performance over longer periods, particularly for the three perennial species (*M. cardinalis*, *M. verbenaceus*, and *M. eastwoodiae*) that were at earlier life stages relative to the annuals during the experiment. Although *RGR* is related to flower number in several of these species (Weimer A., Sheth S., Unpublished data), it would be valuable to study niche breadth partitioning over longer periods and include multiple performance metrics in future work. Finally, since thermal breadth can vary with ontogeny (Carscadden et al., 2020; Donohue et al., 2010; Müller et al., 2018), our findings could differ at later life stages. For instance, niche breadth has been shown to expand with age (Pandori & Sorte, 2019; Parish & Bazzaz, 1985) and size (Gravel et al., 2013). In contrast, thermal niche breadth was narrower for bromeliad growth than germination (Müller et al., 2018).

## 5 CONCLUSIONS

We re‐analyzed a published dataset (Sheth & Angert, 2014a) to illustrate the value of investigating the hierarchical nature of niche breadth, particularly among individuals within populations. In 10 species of western North American monkeyflowers, we showed that populations achieved wide niches primarily via genotypes with divergent environmental optima, and to a lesser extent, general‐purpose genotypes. Genetic variation for niche breadth did not contribute to population‐level niche breadth. Since the niche breadths of individual genotypes encompassed at least 60% of the total niche breadths of their respective populations, the genotype‐level environmental tolerance should often provide a reasonable surrogate for the relative vulnerability or resilience of each population in the face of changing climate (Charmantier et al., 2008). Given that populations with broad environmental tolerances consisted of generalist individuals with greater genetic divergence in environmental optima, these populations may be doubly buffered from environmental change, whereas populations consisting of individuals with narrow tolerances and less divergence in environmental optima may be jeopardized. Future studies considering additional biological hierarchies will provide more comprehensive insights into how the partitioning of niche breadth among individuals, populations, and species will shape biotic responses to climate change.

## AUTHOR CONTRIBUTIONS

**Aeran O. Coughlin:** Formal analysis (lead); visualization (lead); writing – original draft (equal); writing – review and editing (supporting). **Rachel Wooliver:** Conceptualization (supporting); formal analysis (equal); visualization (equal); writing – original draft (supporting); writing – review and editing (supporting). **Seema N. Sheth:** Conceptualization (lead); project administration (lead); resources (lead); supervision (lead); writing – original draft (equal); writing – review and editing (lead).

## CONFLICT OF INTEREST

The authors of this manuscript have no conflicts of interest to declare.

## Supporting information


Supporting Information

## Data Availability

All data associated with this manuscript were previously published at https://doi.org/10.5061/dryad.2b906, and all R scripts are available at https://github.com/emcoughlin/mimulus‐breadth‐partitioning.
