# Supplementary material for: Populations of western North American monkeyflowers accrue niche breadth primarily via genotypic divergence in environmental optima
Source: Ecol Evol. 2022 Oct 22;12(10):e9434. doi: 10.1002/ece3.9434 (PMC9587463; doi:10.1002/ece3.9434)
Supplement: Supplementary file 1 — Supporting Information [file ECE3-12-e9434-s001.pdf]

Supporting Information for

General-purpose genotypes with divergent niche optima shape population-level niche breadth in western North American monkeyflowers

Table S1. Thermal breadth truncation rates across populations. To prevent extrapolation of $T_{breadth}$, if either the lower or upper bound of $T_{breadth}$ fell outside of the temperature measurement interval (i.e., below 15°C or above 50°C), it was given the value of the closest measurement temperature. Values represent truncation rates, averaged among individuals within each population.

Species	Mean proportion of lower bounds truncated	Mean proportion of upper bounds truncated
M. bicolor	0	0.005
M. cardinalis	0.038	0.421
M. eastwoodiae	0.277	0.001
M. filicaulis	0	0
M. floribundus	0.001	0.001
M. guttatus	0	0.021
M. laciniatus	0	0.001
M. norrisii	0	0
M. parishii	0.02	0.288
M. verbenaceus	0.509	0.017

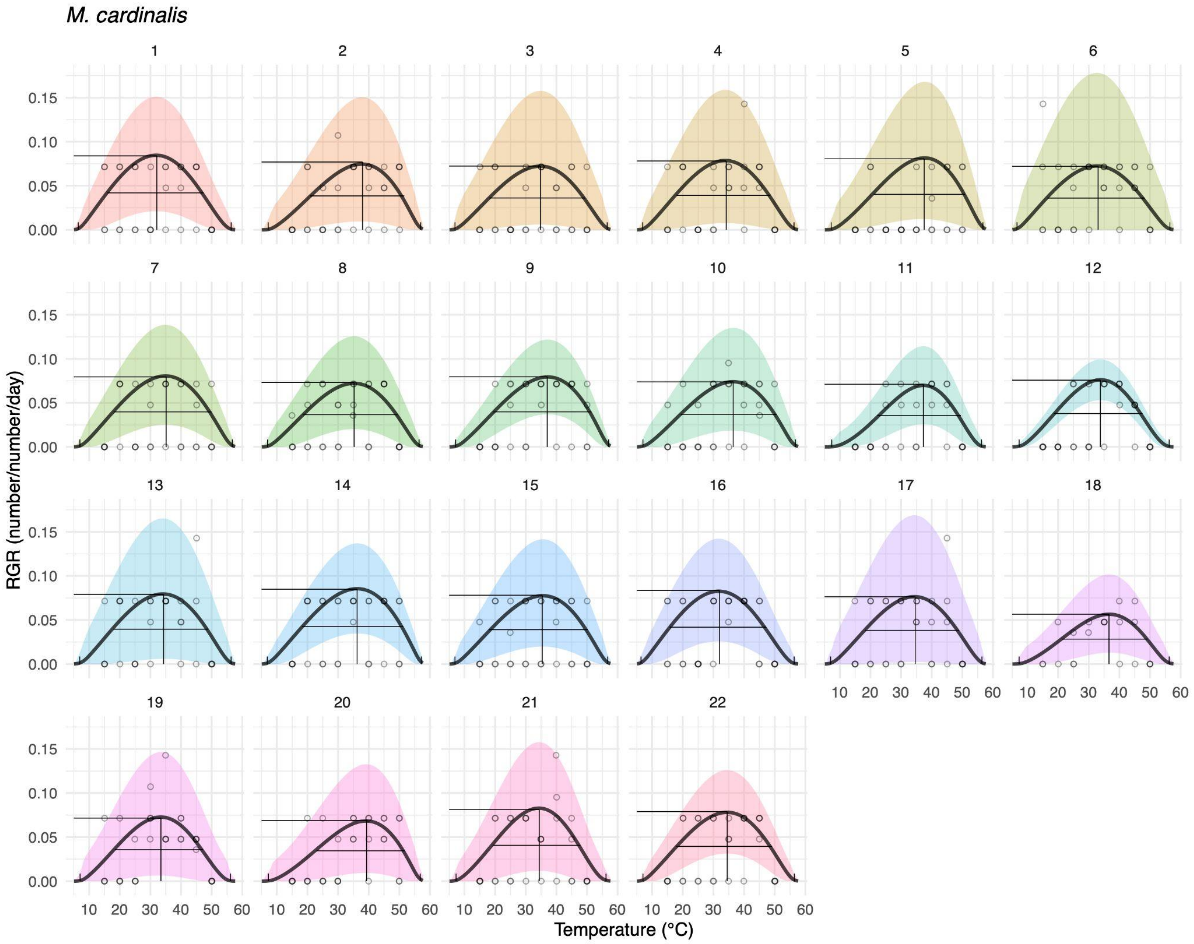

Figure S1. Fitted thermal performance curves (solid black lines) for each family (represented by unique numbers) of *Mimulus cardinalis*. Vertical lines represent thermal optimum, horizontal lines within each curve represent thermal breadth (range of temperatures across which plants achieve 50% of maximum growth), horizontal lines from each curve's peak to the y-axis represent maximum performance, and notches on the x-axis indicate lower and upper thermal limits. The x-axis represents daytime temperatures in growth chambers. Open points represent observed relative growth rate (*RGR*) for each individual plant at each temperature, and shaded colors represent 95% credible intervals for each thermal performance curve.

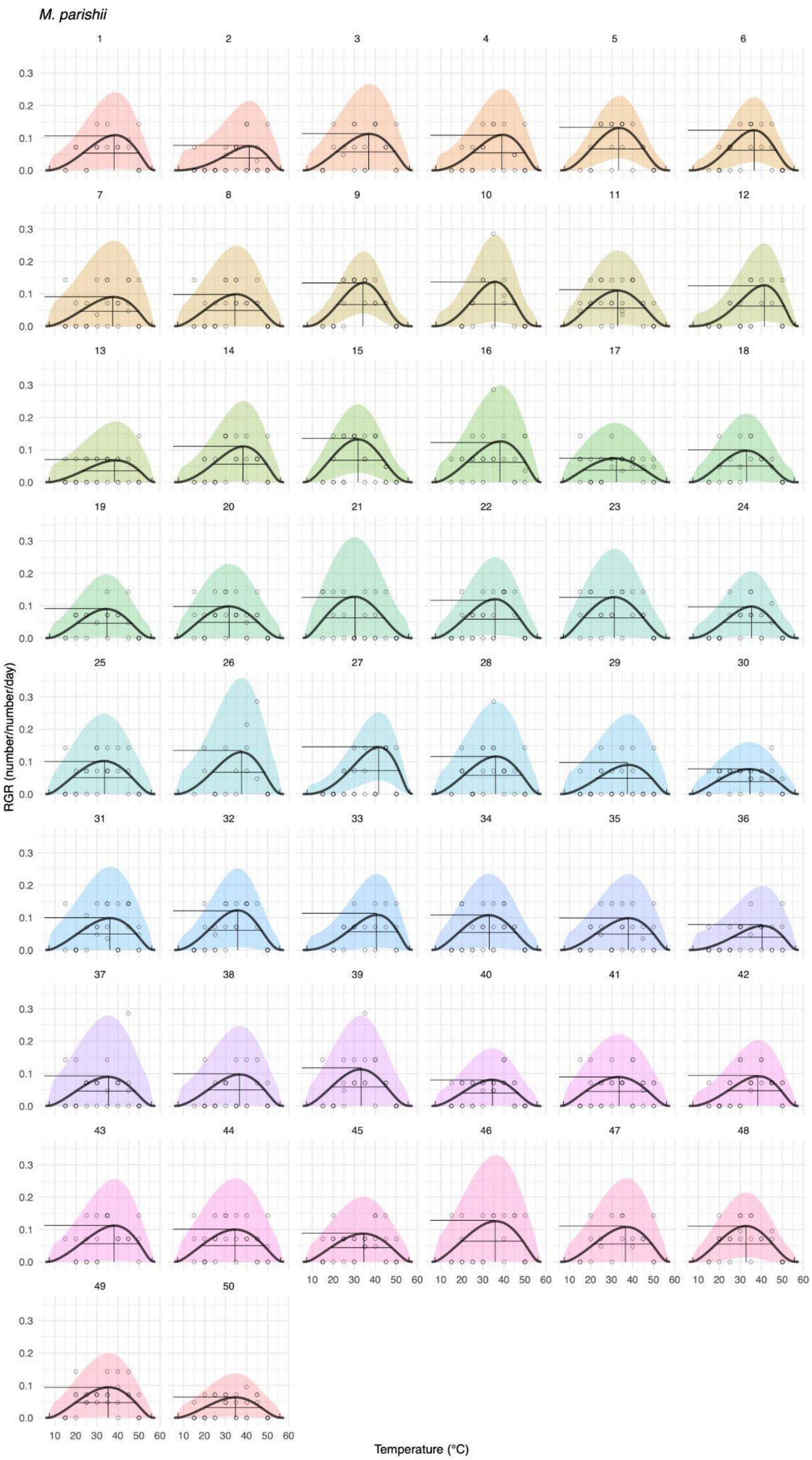
M. parishii
RGR (number/number/day)
Temperature (°C)

Figure S2. Fitted thermal performance curves (solid black lines) for each family (represented by unique numbers) of *Mimulus parishii*. Vertical lines represent thermal optimum, horizontal lines within each curve represent thermal breadth (range of temperatures across which plants achieve 50% of maximum growth), horizontal lines from each curve's peak to the y-axis represent maximum performance, and notches on the x-axis indicate lower and upper thermal limits. The x-axis represents daytime temperatures in growth chambers. Open points represent observed relative growth rate (*RGR*) for each individual plant at each temperature, and shaded colors represent 95% credible intervals for each thermal performance curve.

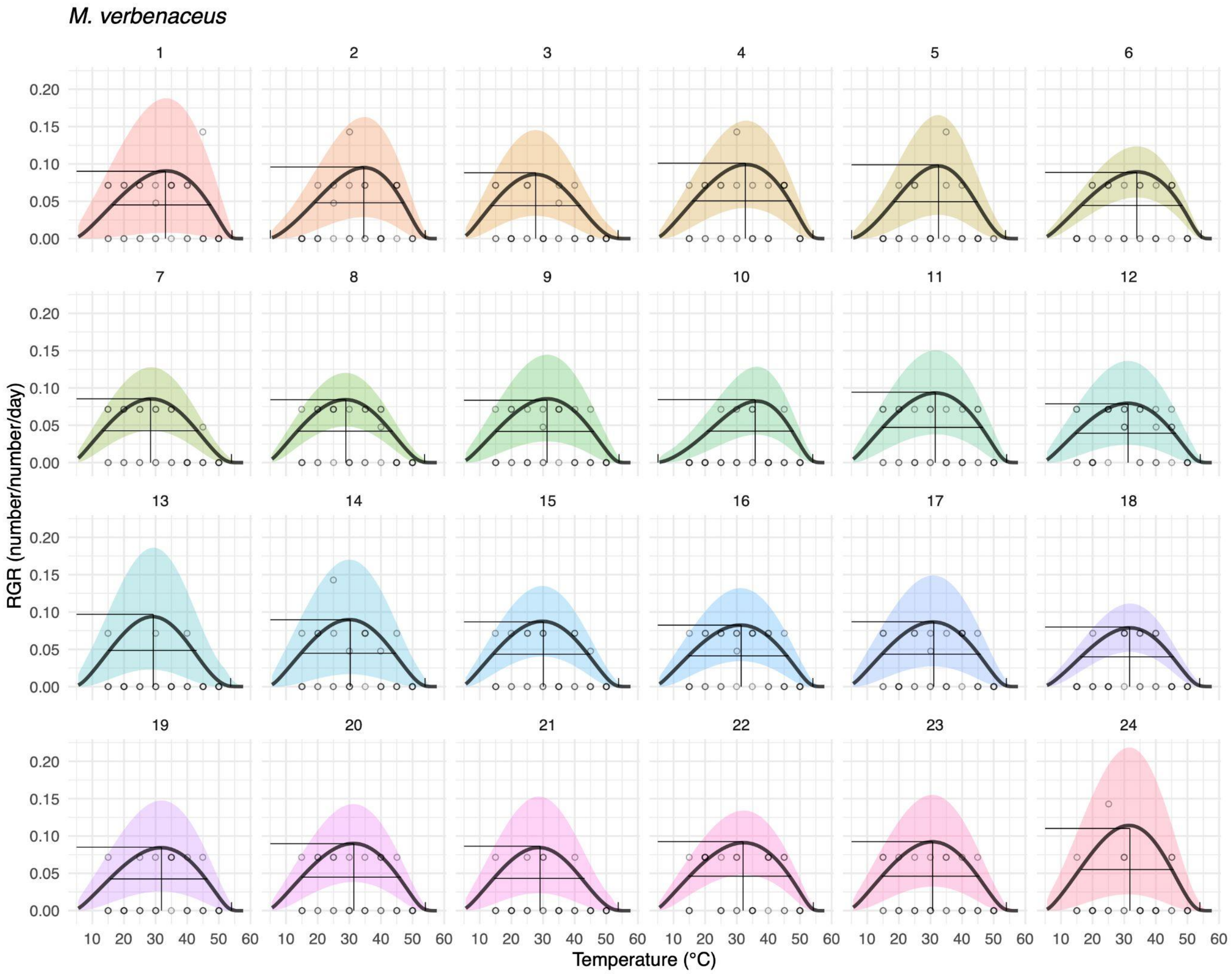

Figure S3. Fitted thermal performance curves (solid black lines) for each family (represented by unique numbers) of *Mimulus verbenaceus*. Vertical lines represent thermal optimum, horizontal lines within each curve represent thermal breadth (range of temperatures across which plants achieve 50% of maximum growth), horizontal lines from each curve's peak to the y-axis represent maximum performance, and notches on the x-axis indicate lower and upper thermal limits. The x-axis represents daytime temperatures in growth chambers. Open points represent observed relative growth rate (*RGR*) for each individual plant at each temperature, and shaded colors represent 95% credible intervals for each thermal performance curve.

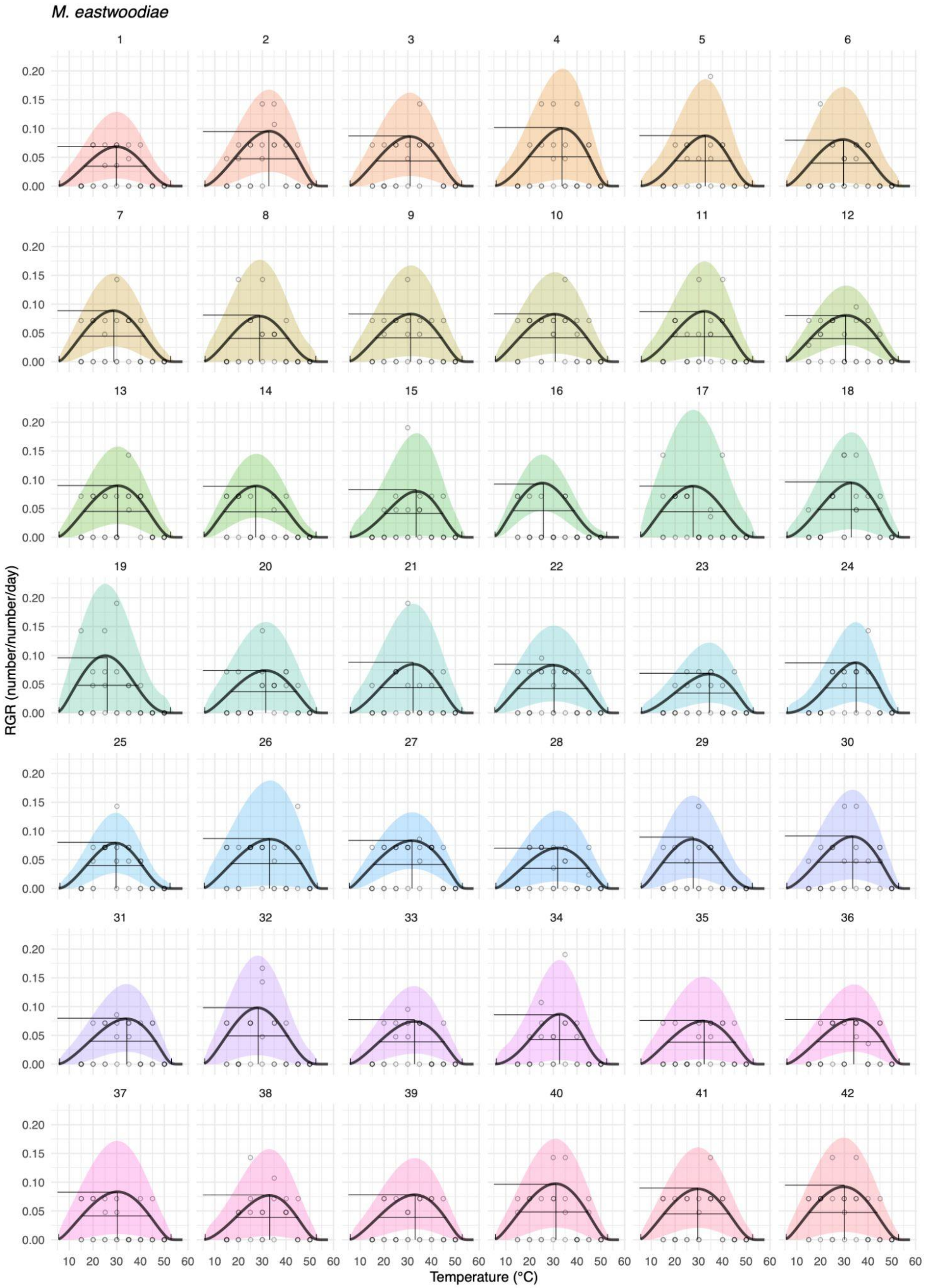

M. eastwoodiae
RGR (number/number/day)
Temperature (°C)

Figure S4. Fitted thermal performance curves (solid black lines) for each family (represented by unique numbers) of *Mimulus eastwoodiae*. Vertical lines represent thermal optimum, horizontal lines within each curve represent thermal breadth (range of temperatures across which plants achieve 50% of maximum growth), horizontal lines from each curve's peak to the y-axis represent maximum performance, and notches on the x-axis indicate lower and upper thermal limits. The x-axis represents daytime temperatures in growth chambers. Open points represent observed relative growth rate (RGR) for each individual plant at each temperature, and shaded colors represent 95% credible intervals for each thermal performance curve.

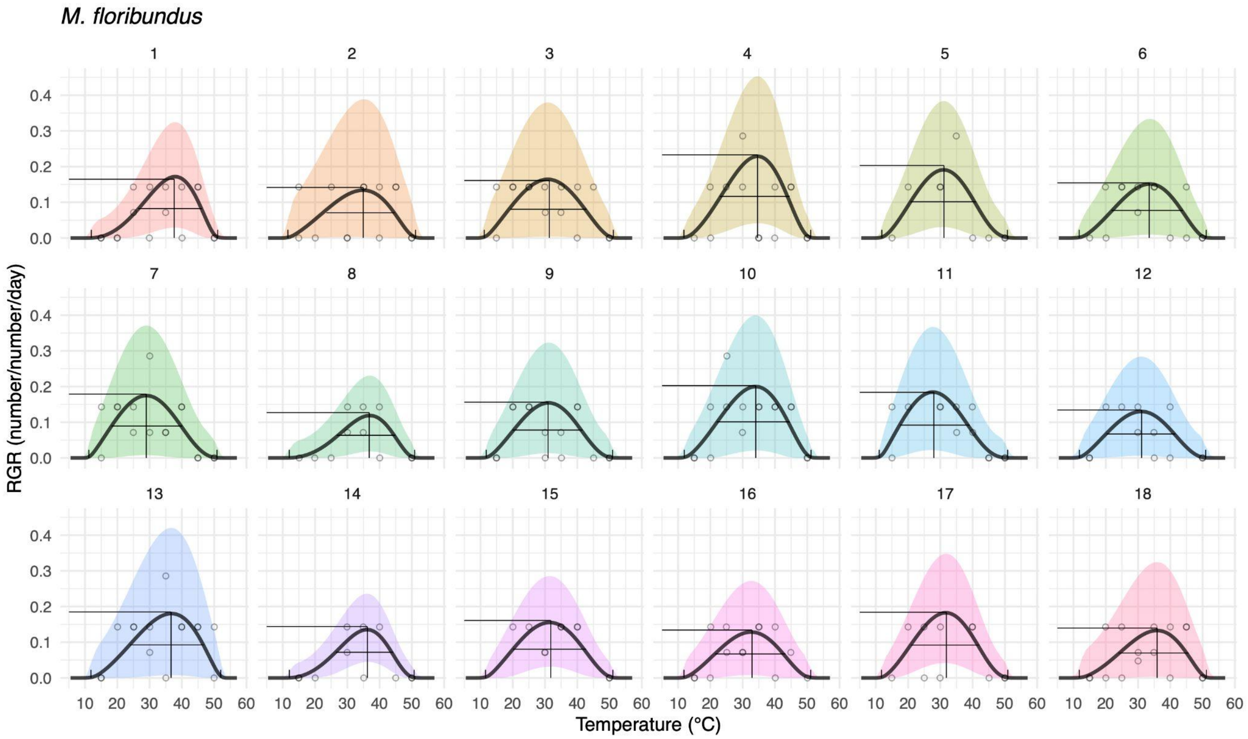

Figure S5. Fitted thermal performance curves (solid black lines) for each family (represented by unique numbers) of *Mimulus floribundus*. Vertical lines represent thermal optimum, horizontal lines within each curve represent thermal breadth (range of temperatures across which plants achieve 50% of maximum growth), horizontal lines from each curve's peak to the y-axis represent maximum performance, and notches on the x-axis indicate lower and upper thermal limits. The x-axis represents daytime temperatures in growth chambers. Open points represent observed relative growth rate (*RGR*) for each individual plant at each temperature, and shaded colors represent 95% credible intervals for each thermal performance curve.

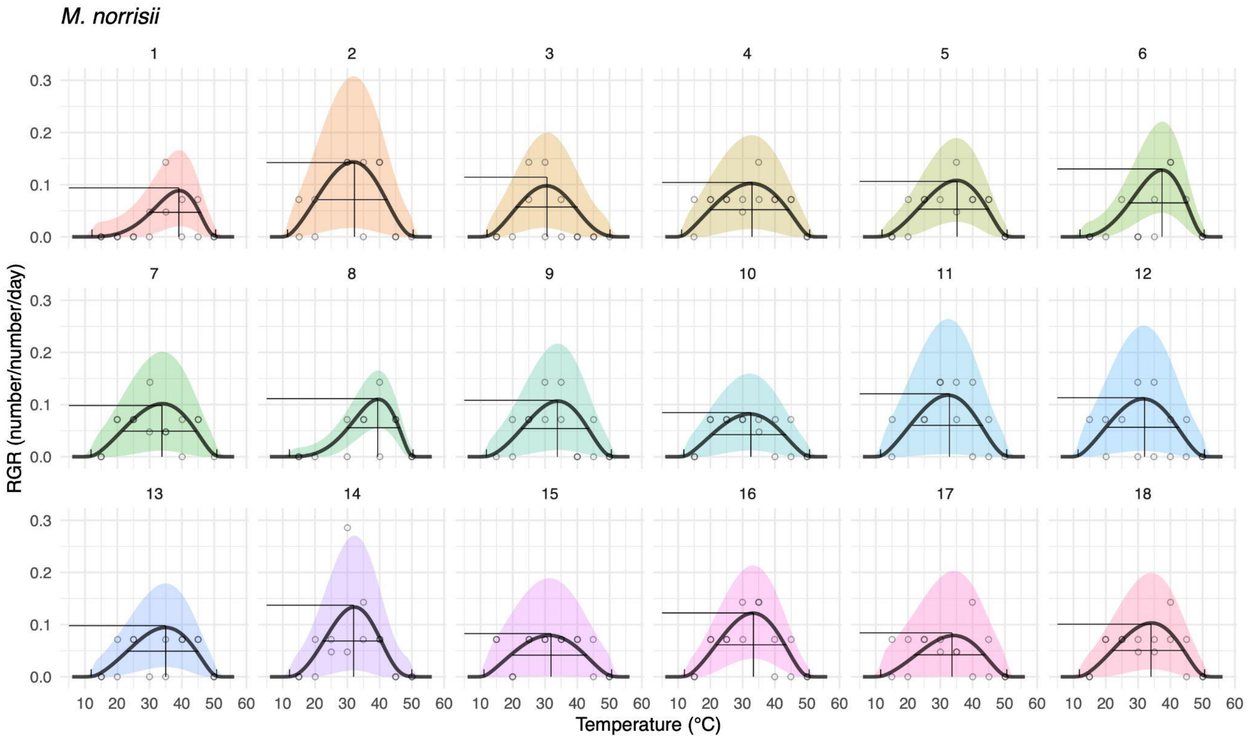

Figure S6. Fitted thermal performance curves (solid black lines) for each family (represented by unique numbers) of *Mimulus norrisii*. Vertical lines represent thermal optimum, horizontal lines within each curve represent thermal breadth (range of temperatures across which plants achieve 50% of maximum growth), horizontal lines from each curve's peak to the y-axis represent maximum performance, and notches on the x-axis indicate lower and upper thermal limits. The x-axis represents daytime temperatures in growth chambers. Open points represent observed relative growth rate (*RGR*) for each individual plant at each temperature, and shaded colors represent 95% credible intervals for each thermal performance curve.

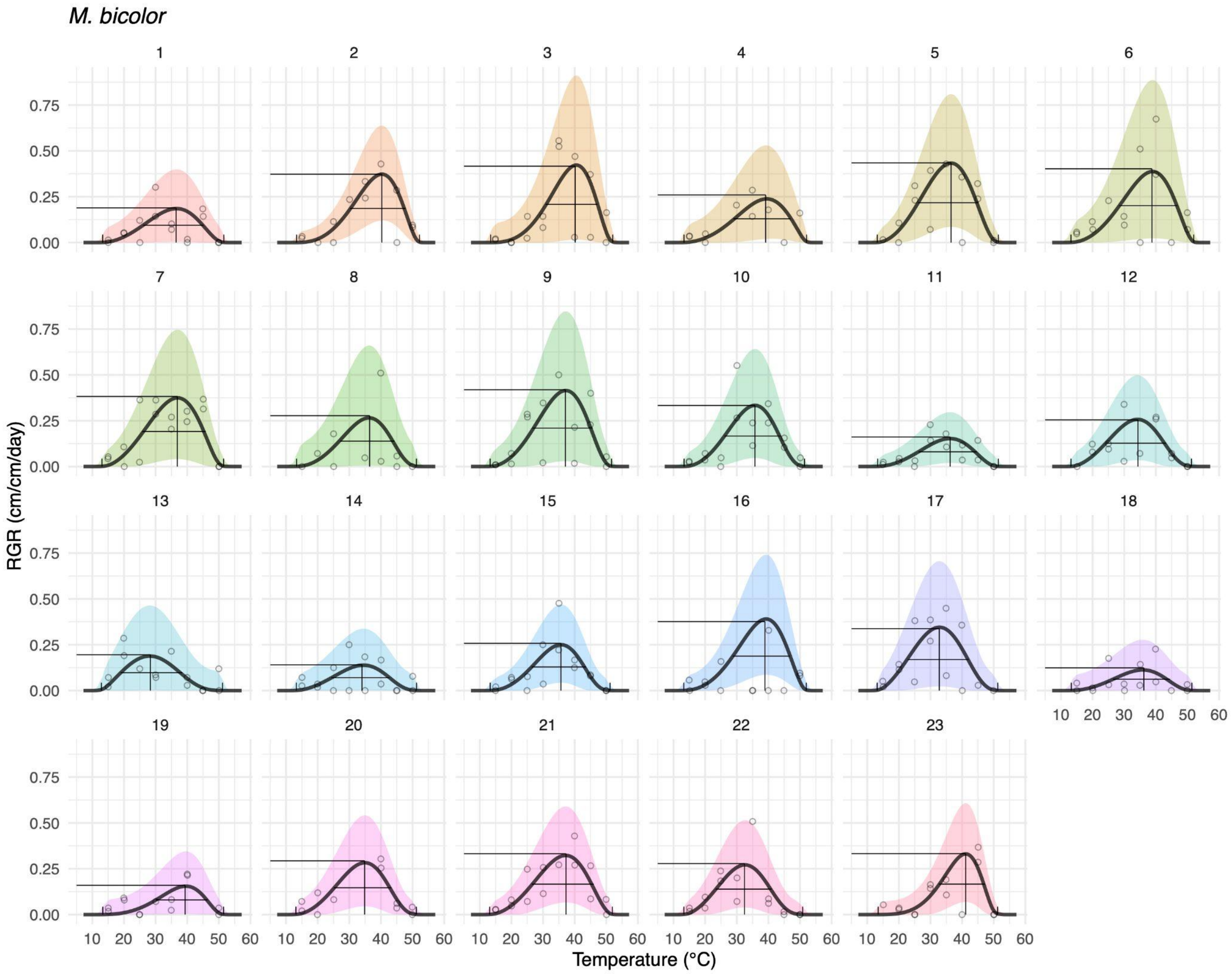

Figure S7. Fitted thermal performance curves (solid black lines) for each family (represented by unique numbers) of *Mimulus bicolor*. Vertical lines represent thermal optimum, horizontal lines within each curve represent thermal breadth (range of temperatures across which plants achieve 50% of maximum growth), horizontal lines from each curve's peak to the y-axis represent maximum performance, and notches on the x-axis indicate lower and upper thermal limits. The x-axis represents daytime temperatures in growth chambers. Open points represent observed relative growth rate (*RGR*) for each individual plant at each temperature, and shaded colors represent 95% credible intervals for each thermal performance curve.

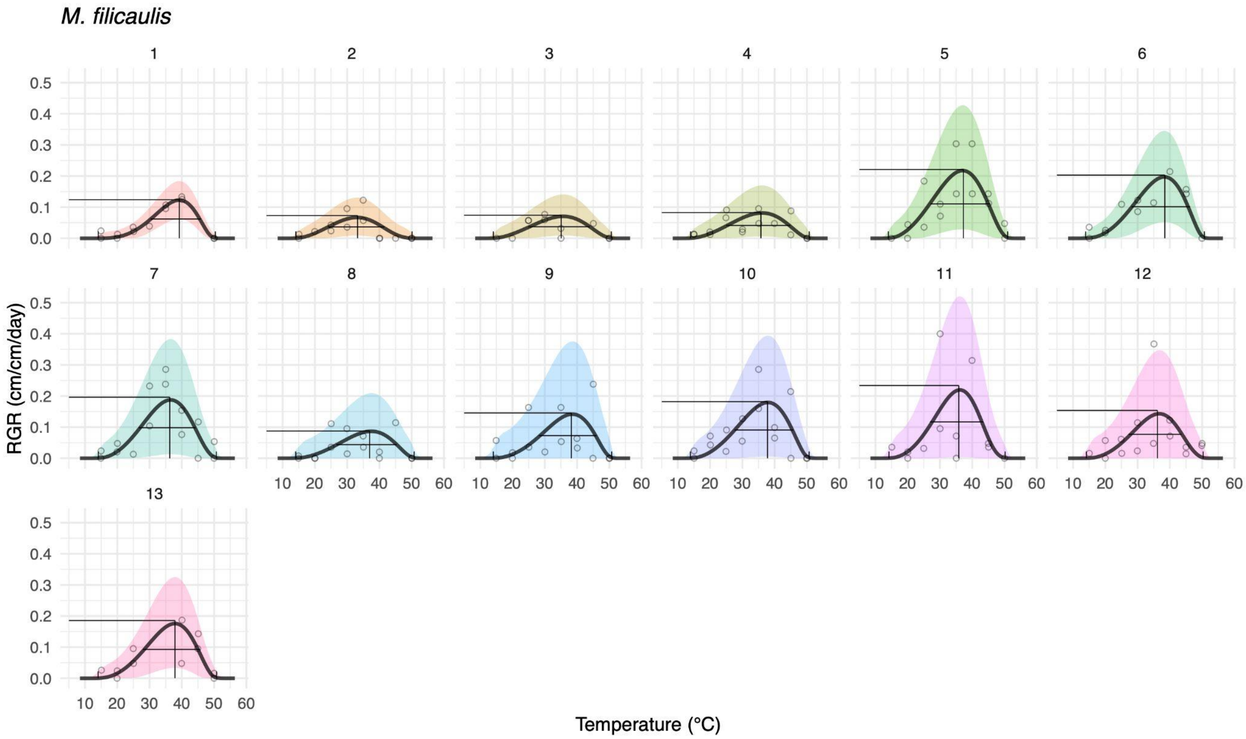

Figure S8. Fitted thermal performance curves (solid black lines) for each family (represented by unique numbers) of *Mimulus filicaulis*. Vertical lines represent thermal optimum, horizontal lines within each curve represent thermal breadth (range of temperatures across which plants achieve 50% of maximum growth), horizontal lines from each curve's peak to the y-axis represent maximum performance, and notches on the x-axis indicate lower and upper thermal limits. The x-axis represents daytime temperatures in growth chambers. Open points represent observed relative growth rate (*RGR*) for each individual plant at each temperature, and shaded colors represent 95% credible intervals for each thermal performance curve.

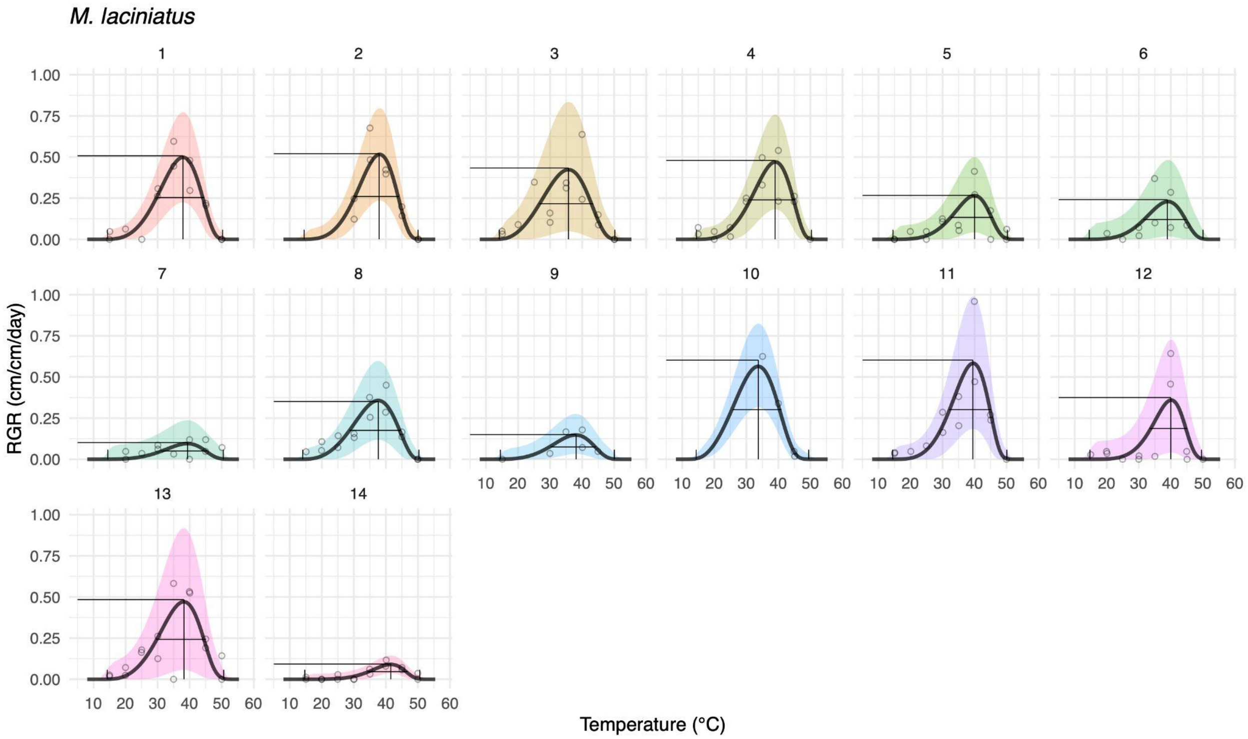

Figure S9. Fitted thermal performance curves (solid black lines) for each family (represented by unique numbers) of *Mimulus laciniatus*. Vertical lines represent thermal optimum, horizontal lines within each curve represent thermal breadth (range of temperatures across which plants achieve 50% of maximum growth), horizontal lines from each curve's peak to the y-axis represent maximum performance, and notches on the x-axis indicate lower and upper thermal limits. The x-axis represents daytime temperatures in growth chambers. Open points represent observed relative growth rate (*RGR*) for each individual plant at each temperature, and shaded colors represent 95% credible intervals for each thermal performance curve.

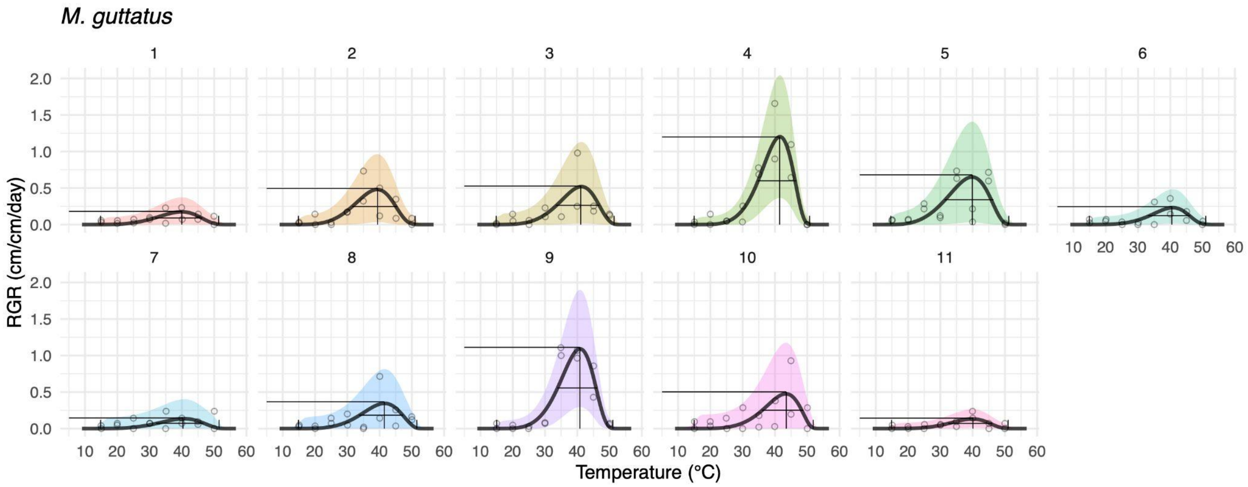

Figure S10. Fitted thermal performance curves (solid black lines) for each family (represented by unique numbers) of *Mimulus guttatus*. Vertical lines represent thermal optimum, horizontal lines within each curve represent thermal breadth (range of temperatures across which plants achieve 50% of maximum growth), horizontal lines from each curve's peak to the y-axis represent maximum performance, and notches on the x-axis indicate lower and upper thermal limits. The x-axis represents daytime temperatures in growth chambers. Open points represent observed relative growth rate (*RGR*) for each individual plant at each temperature, and shaded colors represent 95% credible intervals for each thermal performance curve.
